# An Ancestral Major Histocompatibility Complex Organization in Cartilaginous Fish: Reconstructing MHC Origin and Evolution

**DOI:** 10.1093/molbev/msad262

**Published:** 2023-12-06

**Authors:** Ana Veríssimo, L Filipe C Castro, Antonio Muñoz-Mérida, Tereza Almeida, Arnaud Gaigher, Fabiana Neves, Martin F Flajnik, Yuko Ohta

**Affiliations:** CIBIO-InBIO, Research Center in Biodiversity and Genetic Resources, University of Porto, Vairão 4485-661, Portugal; BIOPOLIS Program in Genomics, Biodiversity and Land Planning, CIBIO, Vairão 4485-661, Portugal; Department of Biology, Faculty of Sciences, University of Porto, Porto 4169-007, Portugal; CIIMAR, Centro Interdisciplinar de Investigação Marinha e Ambiental, Universidade do Porto, Matosinhos, Portugal; CIBIO-InBIO, Research Center in Biodiversity and Genetic Resources, University of Porto, Vairão 4485-661, Portugal; BIOPOLIS Program in Genomics, Biodiversity and Land Planning, CIBIO, Vairão 4485-661, Portugal; CIBIO-InBIO, Research Center in Biodiversity and Genetic Resources, University of Porto, Vairão 4485-661, Portugal; BIOPOLIS Program in Genomics, Biodiversity and Land Planning, CIBIO, Vairão 4485-661, Portugal; CIBIO-InBIO, Research Center in Biodiversity and Genetic Resources, University of Porto, Vairão 4485-661, Portugal; BIOPOLIS Program in Genomics, Biodiversity and Land Planning, CIBIO, Vairão 4485-661, Portugal; Research Group for Evolutionary Immunogenomics, Max Planck Institute for Evolutionary Biology, Plön, Germany; Research Unit for Evolutionary Immunogenomics, Department of Biology, University of Hamburg, Hamburg, Germany; CIBIO-InBIO, Research Center in Biodiversity and Genetic Resources, University of Porto, Vairão 4485-661, Portugal; BIOPOLIS Program in Genomics, Biodiversity and Land Planning, CIBIO, Vairão 4485-661, Portugal; Department of Microbiology and Immunology, University of Maryland School of Medicine, Baltimore, MD, USA; Department of Microbiology and Immunology, University of Maryland School of Medicine, Baltimore, MD, USA

**Keywords:** adaptive immune system, antigen receptors, jawed vertebrate, evolution, Elamobranchs, Cartilaginous fish

## Abstract

Cartilaginous fish (sharks, rays, and chimeras) comprise the oldest living jawed vertebrates with a mammalian-like adaptive immune system based on immunoglobulins (Ig), T-cell receptors (TCRs), and the major histocompatibility complex (MHC). Here, we show that the cartilaginous fish “adaptive MHC” is highly regimented and compact, containing (i) a classical MHC class Ia (MHC-Ia) region containing antigen processing (antigen peptide transporters and immunoproteasome) and presenting (MHC-Ia) genes, (ii) an MHC class II (MHC-II) region (with alpha and beta genes) with linkage to *beta-2-microglobulin* (*β2m*) and *bromodomain-containing 2*, (iii) nonclassical MHC class Ib (MHC-Ib) regions with 450 million-year-old lineages, and (iv) a *complement C4* associated with the MHC-Ia region. No MHC-Ib genes were found outside of the elasmobranch MHC. Our data suggest that both MHC-I and MHC-II genes arose after the second round of whole-genome duplication (2R) on a human chromosome (huchr) 6 precursor. Further analysis of MHC paralogous regions across early branching taxa from all jawed vertebrate lineages revealed that Ig/TCR genes likely arose on a precursor of the huchr9/12/14 MHC paralog. The *β2m* gene is linked to the Ig/TCR genes in some vertebrates suggesting that it was present at 1R, perhaps as the donor of C1 domain to the primordial MHC gene. In sum, extant cartilaginous fish exhibit a conserved and prototypical MHC genomic organization with features found in various vertebrates, reflecting the ancestral arrangement for the jawed vertebrates.

## Introduction

**Fig. 1.**
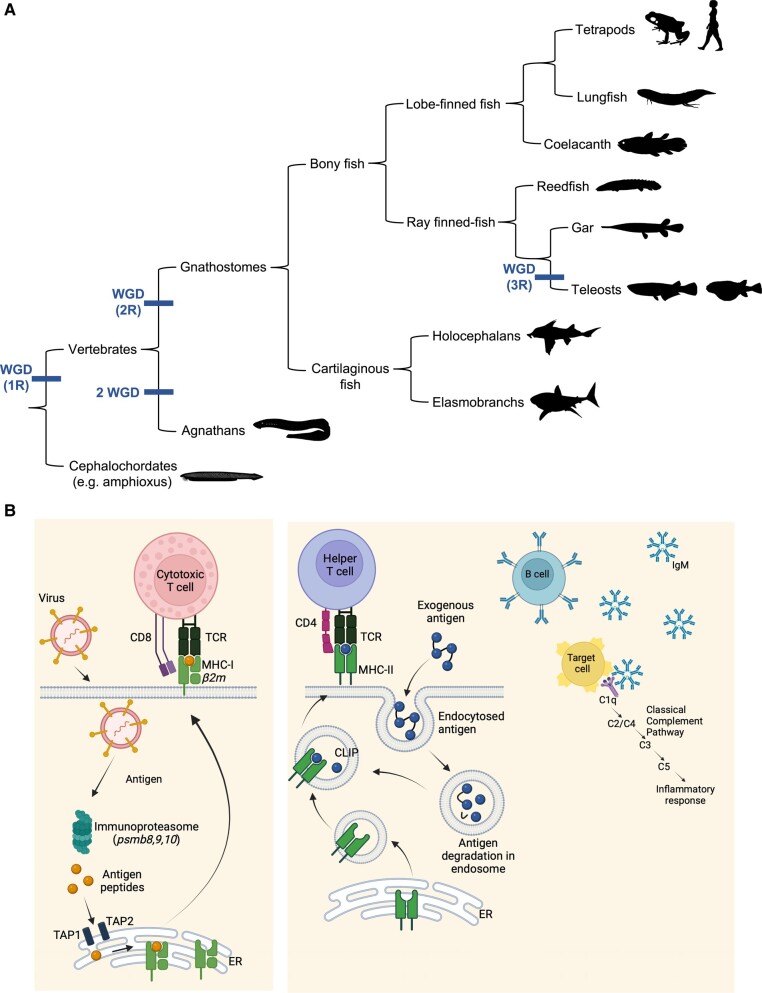
Schematic diagram of vertebrate phylogenetic relationships a) showing the major jawed vertebrate lineages and the putative timings of whole genome duplication (WGD) events. Simplified view of antigen processing and presentation by MHC-I and MHC-II to TCR b), showing also activation of the classical complement pathway by Ig bound to antigen. ER, endoplasmic reticulum; TAP, antigen peptide transporter.

The vertebrate adaptive immune system (AIS) is a complex system of molecules and cells that offer highly specific responses in the fight against pathogens (e.g. toxins, viruses, bacteria, eukaryotic parasites, and cancer cells). The hallmark features of mammalian adaptive immunity include specialized cells (lymphocytes: B and T cells) and their receptors (immunoglobulins [Ig] and T-cell receptors [TCRs], respectively) and ligands (major histocompatibility complex [MHC]; Fig. 1). These features are present in all jawed vertebrates (gnathostomes), from cartilaginous fishes to mammals. The most basal vertebrate lineage, the jawless fishes (agnathans or cyclostomes, i.e. lampreys and hagfishes) also have an AIS based on lymphocytes but with distinct antigen receptors (variable lymphocyte receptors) based on leucine-rich repeats. Agnathans, however, have neither MHC class I (MHC-I)/class II (MHC-II) genes nor the genes involved in antigen processing (e.g. immunoproteasome and antigen peptide transporter genes, *TAP1/2*; Flajnik 2018; Sutoh and Kasahara 2020). Despite its important biological function and impact on individual fitness, the origin and evolution of the AIS are far from understood (Flajnik and Kasahara 2010).

The evolution of the AIS is intimately tied to the emergence and evolution of MHC genes in the jawed vertebrates. These genes are essential for adaptive immune responses, being responsible for antigen presentation to T cells derived from intracellular (e.g. virus, intracellular bacteria via classical MHC Ia [MHC-Ia] genes) and extracellular pathogens (e.g. bacteria, eukaryotic parasites via classical MHC-II genes). Nonclassical class Ib (MHC-Ib) genes have the same structure as MHC-Ia genes but have little or no polymorphism, tissue-specific expression, and/or lack conserved amino acid residues in the antigen-binding pocket; they can thus bind peptide or nonpeptide antigens (e.g. lipids) or may have nonimmune functions. The MHC arose from a putative primordial “proto-MHC” that duplicated twice before the emergence of gnathostomes as part of the 2 whole-genome duplications (WGDs; Fig. 1) that occurred when vertebrates emerged ∼500 million years ago (Dijkstra et al. 2013; Kaufman 2018; Ohta et al. 2019). Indeed, the MHC was one of the first regions to be recognized in which homologous genes are found as syntenic sets in 4 genomic regions in human chromosomes 1, 6, 9, and 19 (i.e. corresponding to the major MHC paralogs MHC*para-1*, -6 [MHC proper], *-9*, and *-19*). Other smaller (minor) genomic regions syntenic to major MHC paralogs in human chromosomes 12, 14, and 15, (i.e. corresponding to the minor MHC paralogs MHC*para-12*, *-14*, and *-15*), but translocated elsewhere during evolution, have also been identified (Flajnik and Kasahara 2010). Neofunctionalization or subfunctionalization of duplicated genes resulted in new sets of genes; e.g. the immunoproteasome genes were generated via duplication of the constitutive catalytic proteasome beta genes (Kasahara et al. 1996). Examination of these MHC paralogous genes and regions allows a prediction of the primordial immune complex (PIC).

Different scenarios of MHC origin and evolution have been proposed to date to reconstruct the sequence of events leading to the current major and minor MHC paralogs (Flajnik and Kasahara 2001; Abi-Rached et al. 2002; Rogers and Kaufman 2016; Ohta et al. 2019). However, MHC structure, diversity, and genomic organization in basal (ectothermic) vertebrates have not been considered in previous studies due to the paucity of available data. Cartilaginous fish (i.e. holocephalans [chimeras and ratfish] and elasmobranchs [sister-lineages of sharks and rays]) are the most basal living jawed vertebrates with an MHC-based AIS, similar to that of mammals (Flajnik 2018). Notably, cartilaginous fishes are an ancient, >450-million-year (Myr)-old lineage of ecologically diverse aquatic organisms (Grogan et al. 2012). They are thus a key group to understand the evolution and perhaps inception of vertebrate adaptive immunity and are essential to identify its ancestral and derived features (Venkatesh et al. 2014; Kaufman 2018).

Chondrichthyan immunobiology and immunogenetics have seen great progress in the last 2 decades, and studies have shown that their immune system is quite complex (reviewed in Smith et al. 2014). Notably, they exhibit a surprising diversity of MHC-I-like genes (i.e. nonclassical MHC-Ib) lineages showing high gene copy number variation and distinct biochemical features at the peptide-binding region (PBR) suggesting the display of different types of antigens and functions (Almeida, Esteves, et al. 2020; Almeida 2021; Almeida et al. 2021). In contrast, nonclassical MHC-II genes (i.e. DM and DO) are lacking in cartilaginous fish (Almeida, Gaigher, et al. 2020) and bony fish (Dijkstra et al. 2013). However, only fragmented data have been reported regarding the genomic architecture of the chondrichthyan MHC (Venkatesh et al. 2014; Zhang et al. 2020).

In recent years, technological advances in next-generation sequencing have allowed the production of high-quality, high-contiguity, whole-genome assemblies (WGAs) in many nonmodel species spanning the jawed vertebrate tree of life. The time is now ripe to perform comparative genomic analyses among basal vertebrates and gain insight into MHC genomic organization and evolution. Here, we performed an in-depth analysis of the elasmobranch MHC to infer its gene composition and genomic architecture, in addition to a comparative genomic analysis of the MHC and paralogous regions across basal living jawed vertebrates (Fig. 1) to gain insights into the origin and evolution of the MHC.

## Results and Discussion

**Fig. 2.**
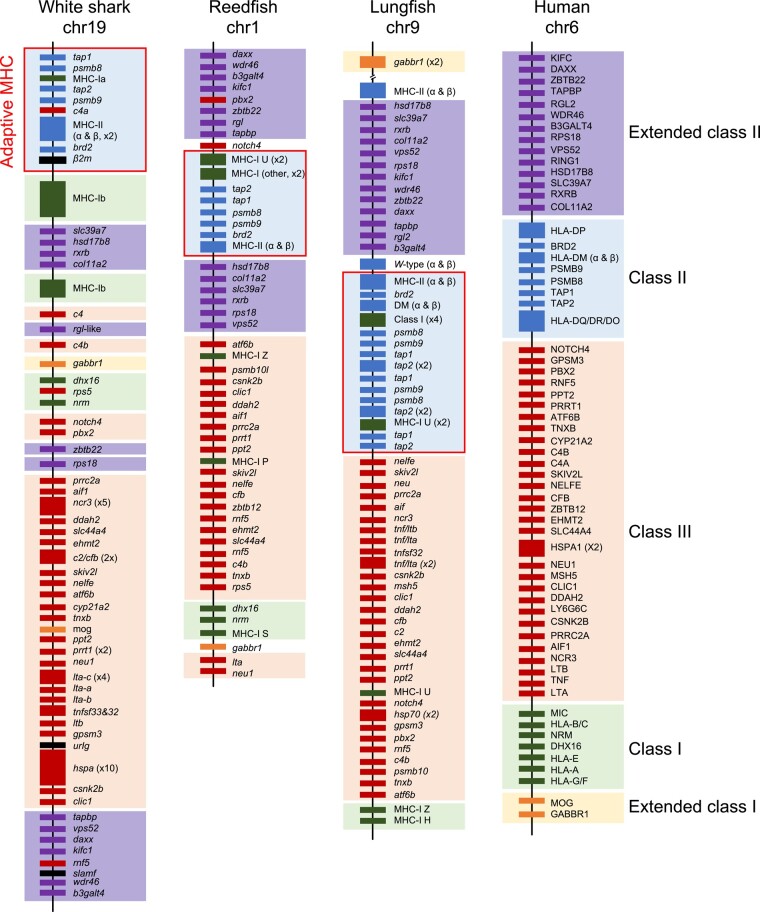
The white shark *C. carcharias* MHC on chromosome 19 has genes orthologous to human MHC-I, -II, and -III but exhibits a unique gene arrangement compared to bony vertebrates, namely reedfish *E. calabaricus* (ray-finned fish) and lungfish *P. annectens* (lobe-finned fish). Gene locations are not to scale. Colored boxes represent the 5 regions of the human MHC used as a reference: extended class II, class II, class III, class I, and extended class I. Genes colored black in white shark are unique to the MHC of elasmobranchs (e.g. *UrIg*) or map outside of the human MHC (e.g. *β2m* and *slamf*). Multicopy genes are shown in wider boxes with copy number in parentheses. Genes from the TNFSF were annotated sensu Redmond et al. (2022).

In mammals, the genomic architecture of the MHC proper has been structured into a class I region including some MHC-Ib genes (often species-specific), a class II region also including nonclassical class II genes and genes responsible for antigen processing of MHC-I proteins (*antigen peptide transporter 1* and *2*, *TAP1/2*; and *proteasome subunit beta type-8* and *9*, PSMB8/9), and an intervening class III region including several immune and nonimmune genes (Fig. 2). This core MHC region is nestled between the extended class I and II regions located at the telomeric and centromeric extremes of the human chromosome 6, respectively. This genomic arrangement has been used for decades as the “gold standard” for MHC genomic comparisons (Kulski et al. 2002).

**Fig. 3.**
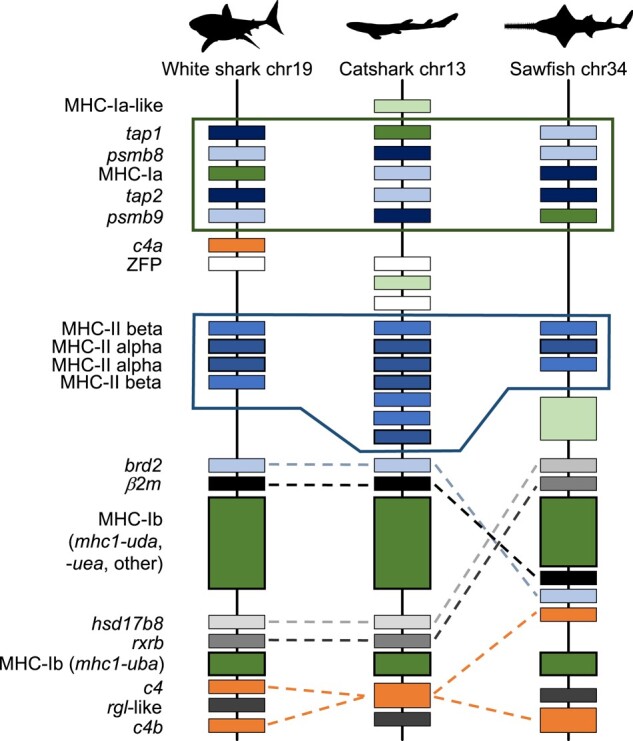
Genomic map showing conserved gene blocks of MHC-Ia (top box) and MHC-II (lower box) regions, in addition to 2 clusters of MHC-Ib genes (dark green with thick black margins) in elasmobranchs, namely in the white shark *C. carcharias*, the catshark *S. canicula*, and the sawfish *P. pectinata*. Genes are color coded using the white shark as reference. Size and distances of genes are not to scale.

Here, we show for the first time that most genes found in the human MHC are also found on a single chromosome in elasmobranchs, i.e. in early branching gnathostomes, but with a distinctive and likely primordial genomic arrangement (Figs. 2 and  3; supplementary files S1 to S3, Supplementary Material online). Most strikingly, we found all MHC-Ib genes to map exclusively near the MHC-Ia genes (closely located to antigen processing genes) and MHC-II genes (Fig. 3) in the elasmobranch genomes—a feature so far exclusive to this group. The next sections provide further details of the elasmobranch MHC and highlight its shared and derived features with other key taxa within the 2 bony fish lineages, the ray-finned (Actinopterygii) and lobe-finned (Sarcopterygii) fish (Fig. 1). For this purpose, we provide new data on the genomic architecture of the MHC for the reedfish *Erpetoichthys calabaricus* and for the lungfish *Protopterus annectens*, i.e. basal taxa within each of these lineages. Our comparative study sheds new light into the genomic arrangement of the MHC in the jawed vertebrate ancestor and its evolutionary path from a PIC.

### A Compact MHC-Ia Region Is Ancestral in Gnathostomes

MHC-Ia molecules bind peptides generated from the degradation of intracellular/cytosolic proteins (e.g. viral proteins) by the immunoproteasome, which are then transported into the endoplasmic reticulum by the heterodimer of TAP1 and 2. We found that WGAs of various elasmobranch species consistently show the close proximity of a single MHC-Ia gene with the antigen processing genes (Fig. 3), namely two of the three catalytically active β-subunits of the immunoproteasome (*psmb8* and *psmb9*) and *tap1* and *2*. Gene order varies according to species, yet all of these genes are located within a region spanning between 87 Kb (sawshark *Pristis pectinata*) and 1.9 Mb (white shark *Carcharodon carcharias*) forming a tight “class Ia region.” These results are consistent with a previous report of another elasmobranch, the nurse shark *Ginglymostoma cirratum* (Ohta et al. 2002).

Such tight linkage of “class Ia region” genes is a common pattern among vertebrates (Fig. 2) with the exception of most placental mammals (Nonaka et al. 1997; Kaufman 1999; Kulski et al. 2002; Zhou et al. 2021), and it has been proposed as a primordial condition of the MHC (reviewed in Ohta and Flajnik 2015). Our results support this hypothesis and firmly establish the existence of a conserved “class Ia region” linking antigen processing and presentation genes in basal gnathostomes. Since the presentation of antigens by MHC-Ia proteins is intimately tied to the functions of the immunoproteasome and *tap*1/2, such a condition would allow for coevolution of these functionally related but structurally distinct genes (Kaufman 1999; Ohta and Flajnik 2015). Indeed, coevolution of *tap* and class Ia genes has been shown for the chicken (Kaufman et al. 1999) and for closely linked *tap2* and MHC-Ia genes in rat (Joly et al. 1998), allowing for the transport of peptide antigens with specific binding affinities to particular class I lineages.

Notably, we found only two of the three genes coding the catalytic β-subunits of the immunoproteasomes (*psmb8* and *psmb9*) in the elasmobranch “class Ia region.” The third gene, *psmb10* (or *mecl1*), was found to map outside the MHC but showed conserved synteny across elasmobranch taxa (supplementary fig. S1, Supplementary Material online). We suggest that this condition is derived rather than ancestral among jawed vertebrates as all 3 genes are found in the MHC in other gnathostome lineages. Yet, the relative location of *psmb10* in the MHC varies between taxa. Indeed, *psmb10* maps in the class III region in many bony fish, both in basal ray-finned fish (e.g. reedfish and bowfin *Amia calva*) and in lobe-finned fish (lungfish and frog *Xenopus tropicalis*) (Fig. 2; Thompson et al. 2021; Ohta et al. 2006), while it maps next to *psmb8* and *9* genes in the class Ia region in many teleosts (e.g. Matsuo et al. 2002; Shiina et al. 2005; McConnell et al. 2016) and in holocephalans (supplementary fig. S2, Supplementary Material online). Inconsistent mapping location and movement out of the MHC of *psmb10* in different jawed vertebrate taxa may be due to its conserved catalytic function as compared to its constitutive paralog (Ferrington and Gregerson 2012), rendering it superfluous in the adaptive immune gene cluster.

Among elasmobranch taxa, we found additional MHC-Ia-like genes close to the MHC-Ia gene (Fig. 2; unpublished data). However, the number of genes differs depending on the species examined. One common feature across the surveyed shark and ray genomes is that these MHC-Ia-like genes are surrounded by multiple copies of zinc finger protein genes, possibly inhibiting recombination between them and the tightly linked bona fide MHC-Ia gene.

### Ancestral Linkage of MHC-Ia and MHC-II Regions Is Supported by Cartilaginous Fish Genomes

MHC-II molecules are heterodimers of alpha and beta proteins encoded by separate genes, which are usually in close proximity in the genome. Our analyses show the existence of a “class II region” in elasmobranchs including only classical MHC-II alpha and beta genes (with one to four copies each, depending on species), and consistently located adjacent to the “class Ia region” across sharks and rays (Figs. 2 and 3). Classical MHC-II genes exhibit divergent allelic lineages in sharks dating back >350 Myr (reviewed in Almeida, Gaigher, et al. 2020). As mentioned above, the nonclassical MHC-II genes DM and DO are absent in cartilaginous fish (Almeida, Gaigher, et al. 2020).

The close location between MHC-II genes and a “class Ia region” found here in elasmobranchs is also present in basal bony vertebrates (Fig. 2), both in the ray-finned (i.e. bowfin, Thompson et al. 2021; reedfish, supplementary file S4, Supplementary Material online) and the lobe-finned (lungfish, supplementary file S5, Supplementary Material online) fish lineages, strongly supporting an ancestral linkage of MHC-Ia and MHC-II genes in jawed vertebrates. This organization is retained in amphibians (Ohta et al. 2006; Ohta et al. 2019), birds (Kaufman and Salomonsen 1997; Kulski et al. 2002), and prototherian mammals (Belov et al. 2006), while MHC-Ia genes are translocated to the telomere of the same chromosome in placental mammals (e.g. the human class I region, Fig. 2). This linkage was lost in teleost fish where MHC-I and MHC-II genes are located on different chromosomes (reviewed in Edholm et al. 2022).

### Ancestral Linkage of *β2m* to the MHC Is Suggested by Cartilaginous Fish Genomes

The *beta-2-microglobulin* (*β2m*) gene encodes for a C1-type Ig superfamily (IgSF) protein that associates with most MHC-I proteins and is essential for their proper protein structure and function. We show here the detailed linkage of MHC-I/-II genes and *β2m* in all elasmobranchs surveyed (and likely in holocephalans), consistent with a common structure of MHC-I/II and *β2m* domains and prediction of the ancestral genetic location in the ancestral MHC (Ohta et al. 2011). Interestingly, the genes syntenic to *β2m* vary among elasmobranch species except for *bromodomain-containing 2* (*brd2*) (Figs. 2 and 3; discussed below). Thus, our findings on the close linkage of the class I and II regions and *β2m* suggest that they form the core of the MHC in elasmobranchs, the ancestral “adaptive MHC,” which is now shown to have emerged in basal gnathostomes as suggested by Ohta and Flajnik (2015). In contrast, the location of *β2m* is variable among bony vertebrates although it is generally not linked to the core MHC (Fig. 2). This pattern has been suggested to be due to the translocation of *β2m* from a primordial location within the MHC. This topic is further examined below in the analysis of MHC paralogous regions.

### A Complement c4 Gene (c4a) Maps to the Prototypical Adaptive MHC

The complement system includes many interacting proteins activated by pathogen-derived molecules resulting in a variety of immune responses bridging innate and adaptive immunity. Among the 3 complement activation pathways, the “classical pathway” is unique to jawed vertebrates and relies on the successive activation of complement proteins C4, C2, C3, and C5 upon binding of C1q to antibodies (e.g. IgM and IgG) bound to pathogens (Nonaka and Smith 2000; Smith and Nonaka 2014). The *c4* and *c2* genes were derived from gene duplication of *c3* and *CFB* genes (as part of the genome-wide duplications), respectively, in the gnathostome ancestor after the divergence of agnathans and gnathostomes (Kimura et al. 2009). *c4* genes are typically found in the MHC-III region in most vertebrates differing in the amino acid residue at the catalytic site: *c4a* bears a catalytic asparagine (D) and preferentially binds to amino groups found in proteins, and c*4b* bears a catalytic histidine (H) and binds primarily to carbohydrates as found on bacterial surfaces. The *c4b*-H is thought to be the ancestral form, like in primordial *c3* proteins (Nonaka et al. 2017).

We found multiple copies of the *c4* gene in the elasmobranch genomes, differing in the amino acid residue at the catalytic site as first noted by Nonaka et al. (2017), and show their genomic location in elasmobranch for the first time. Two of the elasmobranch *c4* genes map closely together, one of which is *c4b*-H and the other has variable residues at the catalytic site (N, S, or R, depending on species) of unknown binding affinities (Fig. 3). Both of these genes map next to the MHC-Ib gene cluster and *Ral guanine nucleotide dissociation stimulator-like* genes (*rgl*-like), as expected, in the class III region. A third *c4* gene, *c4a*-D, is found in the white shark and surprisingly maps within the class Ia region (Figs. 2 and 3). This new finding is remarkable as the *c4a*-D gene preferentially binds protein antigens in Ig-based immune complexes that trigger the classical complement pathway (Nonaka and Smith 2000). The emergence of the *c4* and *c2* genes had the major roles in the emergence of the classical complement pathway; thus, the location of *c4a*-D in or adjacent to elasmobranch MHC-I/-II genes hints at coevolution with key adaptive immune genes. Importantly, our results solidify the connection between the emergence of the gnathostome-specific classical complement pathway and the AIS (Nonaka 2014). The only *c4* genes reported in holocephalans (with catalytic H or A) are closely linked to other genes in the MHC region (Nonaka et al. 2017), suggesting a distinct gene arrangement between cartilaginous fish lineages.

**Fig. 4.**
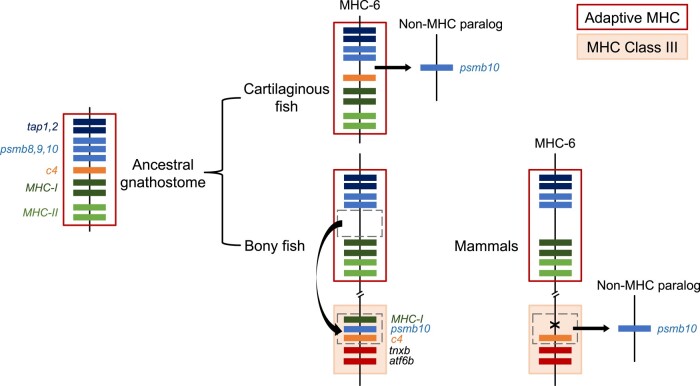
Translocation of *psmb10* and *c4* genes along gnathostome evolution from an ancestral location in the adaptive MHC. Elasmobranchs had *psmb10* translocated out of the MHC into a non-MHC paralogous chromosome. Bony fish had a translocation of the gene block encompassing *psmb10*, *c4*, and MHC-I genes into the class III region. In mammals, an additional translocation of *psmb10* into a non-MHC paralogous chromosome occurred convergently with elasmobranchs.

Mapping of *c4* genes next to the “class Ia region” in elasmobranchs is not observed in other jawed vertebrates, except in birds (Kulski et al. 2002). Thus, it was (so far) thought to be a derived condition unique to birds, in line with the many translocations of MHC genes to other regions of their genomes. However, our analyses on the lungfish genome (Fig. 2) and previous findings in bowfin (Thompson et al. 2021) and *Xenopus* (Ohta et al. 2006) show that the single *c4* gene found in basal ray-finned and lobe-finned fish lineages is next to *psmb10*. Assuming all immunoproteasome genes (*psmb8-10*) were originally in the class Ia region (detailed above), translocation of the *c4b* and *psmb10* block from the adaptive MHC elsewhere on the same chromosome may have occurred after the divergence of cartilaginous and bony fish (Fig. 4). Basal ray-finned fish lend some support to this hypothesis since MHC-I genes are in the vicinity of the *psmb10* gene but away from class Ia/II region in reedfish (our results, Fig. 2) and bowfin (Thompson et al. 2021). Additional translocations of either *c4* or *psmb10* have occurred in vertebrates (e.g. reedfish *c4* away from *psmb10* in the class III region and human *psmb10* onto a non-MHC paralagous chromosome, convergently with elasmobranchs; Fig. 2).

### Nonclassical MHC-Ib Genes of Elasmobranchs Map Exclusively to the MHC

Cartilaginous fish exhibit a large diversity of nonclassical class I (MHC-Ib) lineages of yet unknown functions, some of which are found in both elasmobranchs and holocephalans dating back >450 Myr (Almeida, Esteves, et al. 2020; Almeida 2021; Almeida et al. 2021). Our extensive searches across the surveyed elasmobranch genomes showed that all MHC-Ib genes map to the MHC, adjacent to the MHC-Ia and MHC-II regions (Fig. 3). One large cluster of MHC-Ib genes is found next to the elasmobranch adaptive MHC, including UDA (single copy) and UEA (multicopy) genes, while a second class Ib cluster comprised exclusively of UBA genes (multicopy) is located away from the larger Ib cluster and from the tight “class Ia region” (Fig. 3). Our finding that no MHC-Ia/b or MHC-II genes of elasmobranchs were found outside the equivalent human MHC, on chromosome 6, is exclusive to this group. In fact, such condition contrasts with the patterns observed in all other vertebrate taxa studied to date, including ray-finned and lobe-finned fish, where MHC-Ib genes are found both in the MHC and in other regions of the genome. Our results on the elasmobranch MHC strongly suggest that all MHC genes were originally linked together and had a common origin, in the jawed vertebrate ancestor (more below).

The genes surrounding the elasmobranch MHC-Ib clusters are similar in sharks and rays (as mentioned, the 2 sister lineages of elasmobranchs; Fig. 3). The genes syntenic to 1 cluster of MHC-Ib genes (e.g. UDA and UEA) are conserved in elasmobranchs, including *brd2* and *β2m* on one end and *hydroxysteroid 17-beta dehydrogenase 8* (*hsd17b8*), *retinoid acid receptor beta* (*rxrb*), *solute carrier family 39 member 7* (*slc39a7*), and *collagen alpha-2(XI) chain* (*col11a2*) on the other end (generally considered as extended class II genes; Fig. 3). In turn, a cluster of MHC-Ib UBA genes are flanked by *c4* and *rxrb* genes (mapping in human class III and extended class II regions, respectively). The relative gene location within the “class Ib region” differs between sharks and rays likely due to a genomic inversion that occurred after the split of these elasmobranch sister lineages (Fig. 3).

### W-type Genes Map Outside the MHC in Elasmobranchs

*W*-type MHC genes have been proposed as ancestral to MHC-I and MHC-II genes and have been reported in many jawed vertebrates including cartilaginous fish and both ray-finned and lobe-finned fish (Okamura et al. 2021). These genes share interdomain sequence features with MHC-I genes but exhibit other features typical of MHC-II genes, namely a protein structure with only 2 ectodomains and the presence of alpha and beta genes located as pairs in the genome and forming heterodimers (Okamura et al. 2021). Our results show that *W*-type alpha and beta genes in the surveyed elasmobranch genomes are multicopy, have conserved synteny (supplementary fig. S3, Supplementary Material online), and map to non-MHC paralogous regions. Our analyses on the lungfish genome show similar observations whereby some *W*-type genes also map to non-MHC paralogs and co-occur with some of the conserved syntenic genes observed in elasmobranchs (supplementary fig. S3, Supplementary Material online). However, we found additional *W*-type genes in the lungfish that *do* map to the MHC, as previously reported in the coelacanth and in ray-finned fish (Okamura et al. 2021). The MHC-linked *W*-type genes map in the same genomic region in the 2 lobe-finned fish taxa and show high amino acid similarity (supplementary figs. S4 and S5, Supplementary Material online). In contrast, our analyses did not detect *W*-type genes in reedfish. Based on our and previous findings, the ancestral location of *W*-type genes in the MHC region is supported by observations in the 2 bony fish lineages, despite evidence of early translocations into other parts of the genome (but not to MHC paralogous regions) in elasmobranchs and lungfish. The data show that if *W*-type genes are truly ancestral to MHC-I/II, their emergence was within the MHC proper and not in a proto-MHC region.

### The Core “Adaptive MHC” Postdates 2R

The new evidence assembled here from the elasmobranch MHC genomic architecture indicates the presence of an ancestral “adaptive MHC” core region early in gnathostome evolution. Specifically, this region would include MHC-Ia and MHC-II genes, in addition to structurally and/or functionally relevant genes for MHC protein structure and function, namely *β2m* and antigen processing genes (*psmb8/9* and *tap1/2)*, and possibly complement *c4* genes (Fig. 3 and region highlighted in red in Fig. 2). Our observations on basal bony fish further suggest that *W*-type MHC genes and the third immunoproteasome gene *psmb10* (as mentioned above) were also part of the adaptive MHC core region in the ancestral gnathostome (as also proposed by Okamura et al. 2021; Kasahara et al. 1996, respectively).

**Fig. 5.**
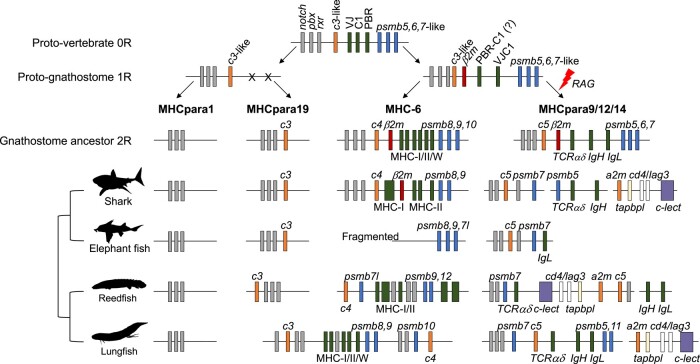
Hypothetical scenario of MHC evolution from a PIC to the four MHC paralogous regions of gnathostomes. The current genomic architecture of the MHC paralogs is depicted from early branching living representatives of all jawed vertebrate lineages, namely cartilaginous fish: white shark *C. carcharias* (elasmobranchs) and elephant fish *C. milii* (holocephalans) and bony fish: reedfish *E. calabaricus* (Actinopterygii, ray-finned fish) and lungfish *P. annectens* (Sarcopterygii, lobe-finned fish). MHC paralogous framework genes (*notch*, *pbx*, and *rxr*) are colored gray; complement genes (*C3*-like, *C3*, *C4*, and *C5*) and *alpha-2 microglobulin* (*a2m*) are colored orange; *tapasin-related protein* (*tapbpl*) is colored light yellow; MHC/Ig/TCR and precursor genes are colored dark green; proteasome genes and precursors are colored blue; *β−2 microglobulin (β2m*) is colored red; *CD4* and *lag3* are colored white; and *C*-type lectins (C-lect) are colored purple. Genes lost from paralogs are represented by X, and gene sizes and distances are not to scale.

This conserved ancestral genomic organization suggests for the first time that the “adaptive MHC” arose only after the second round (2R) of WGD (Fig. 5). Multiple translocation events of descendant MHC genes would have subsequently occurred throughout vertebrate evolution. Previous work on mammals reported MHC-I genes mapping in multiple MHC paralogs (e.g. humans have MHC-Ia in MHC*-6*, and MHC-Ib-*CD1* in MHC*para-1*), thus suggesting MHC inception prior to the first round of WGD (i.e. at 0R; reviewed in Ohta et al. 2019). This scenario was challenged by showing that the *CD1* genes in human MHC*para-1* resulted from an earlier translocation from the MHC*-para6*, thus placing MHC inception after the first round (1R) of WGD (Ohta et al. 2019). Further support for this hypothesis was provided by the presence of *psmb6* with an MHC-Ib (mhc1-uda) in the equivalent major MHC*para-19* of *Xenopus* (Ohta et al. 2019). However, we found that many genes syntenic to *psmb6* are shared among elasmobranchs, lobe-finned fish (i.e. lungfish and humans) and ray-finned fish (i.e. reedfish and spotted gar *Lepisosteus osseus*), and do not include MHC-I genes (supplementary table S3, Supplementary Material online). These observations suggest that the condition in *Xenopus* may be derived and not ancestral. Therefore, we propose that *psmb6* was originally located next to *psmb5* and *psmb7* genes but was translocated into non-MHC paralogs prior to the divergence of cartilaginous and bony fish, in line with Kasahara et al. (1996) and consistent with MHC origin at 2R.

The exception to an MHC origin at 2R is the presence of *mhc1-uea* (MHC-Ib-112) mapping conservatively to the equivalent MHC*para-14* of amphibians and reptiles, again implying MHC-I emergence at 1R. This gene is highly divergent from other MHC-I genes and has been suggested to be of ancient origin (Ohta et al. 2019). Thus far, no *mhc1-uea* has been detected in cartilaginous or ray-finned fish; thus, whether this gene results from a recent translocation from the equivalent MHC*para-6* in tetrapods but lost in birds and mammals (supporting MHC inception at 2R) or from multiple gene loss events in *MHCpara-9* along vertebrate evolution (supporting the alternative hypothesis of MHC inception at 1R) remains to be ascertained.

The presence of MHC-Ia, MHC-II, and *W*-type in the same genomic region of the gnathostome ancestor implies that all MHC molecules would have originated prior to the divergence of cartilaginous and bony fish and, thus, in a relatively “short” time (Fig. 5). This assumption may be further extended to (some) MHC-Ib genes. Recently, we found a new MHC-Ib lineage in elasmobranchs that is very similar to CD1 in terms of its antigen binding region and biophysical features (i.e. MHC-Ib UFA; unpublished data), and that maps in one of the MHC-Ib clusters (including *UDA* and *UEA*; Figs. 2 and 3). While orthology between CD1 and UFA is challenging to assess given the long divergence times between them, the existence of CD1-type MHC-Ib genes in elasmobranchs suggests that some MHC-Ib genes may be as old as MHC-Ia, MHC-II, and *W*-type. This scenario does not exclude the divergence of additional lineage-specific MHC-Ib genes from extant MHC-I genes during gnathostome evolution, similar to MHC-II leading to nonclassical DM in the lobefin fish lineage (Dijkstra et al. 2013).

### Comparative Genomics of MHC Paralogs Suggest a Linked Ancestral MHCpara-9/12/14

Hallmark genes of the 4 major MHC paralogous regions mapped here in elasmobranchs as well as in basal bony vertebrate genomes generally follow the patterns reported in tetrapods: genomic regions matching the equivalent human MHC*para-1*, *-6*, *-9*, and *-19* were found on separate chromosomes (except lungfish; supplementary table S2 and file S5, Supplementary Material online). The minor MHC paralogs, equivalent to human MHC*para-12* (including the natural killer cell complex [NKC] genes) and MHC*para-14* (including *IgH*/*IgL*/*TCRαδ* genes), may be incompletely assembled in elasmobranchs as suggested by the existence of TCR and Ig genes in multiple unplaced scaffolds or the absence of some genes from the available assemblies (e.g. *cd4* and *limphocyte-activation gene 3* [*lag3*] in *P. pectinata*) (supplementary table S1, Supplementary Material online).

Strikingly, our observations from basal bony vertebrates show a different genomic architecture of minor MHC paralogs from tetrapods. Indeed, the minor MHC*para-12* and *-14* showed a consistent association with the major MHC*para-9* hitherto not found in vertebrates. Specifically, in the reedfish *E. calabaricus*, the TCRαδ gene cluster associated with minor huMHC*para-14* is closely located to hallmark genes of huMHC*para-9*, which, in turn, are interspersed with genes generally associated with minor huMHC*para-12* (e.g. *tap-binding protein-like* [*tapbpl*], *lag3*, *cd4*, and *c*-type lectins) (supplementary file S7, Supplementary Material online). Similarly, a single chromosome of lungfish includes all hallmark genes of huMHC*para-14* (i.e. *psmb5/11*, *TCRαδ*, and *IgH*) and huMHC*para-9*, although the latter maps between genes syntenic to minor huMHC*para-14* in tetrapods (supplementary file S8, Supplementary Material online). Interestingly, hallmark genes of huMHC*para-14* and huMHC*para-9* are found in chromosome 1 of the opossum *Monodelphis domestica* (Belov et al. 2007). The linkage of the equivalent hu*MHCpara-9* with minor huMHC*para-12* and *-14* genes in ray-finned and lobe-finned fish suggests that such arrangement may be the ancestral condition in (at least) bony vertebrates.

We found additional evidence further warranting the ancestral link of the equivalent hu*MHCpara-9/12/14* at the origin of gnathostomes. Specifically, 1 scaffold of the holocephalan *Callorhinchus milii* genome assembly has all hallmark genes of huMHC*para-9* in addition to the *IgL λ* gene cluster associated with minor MHC*para-14* (GenBank accession no. NW_024704749.1; supplementary file S9, Supplementary Material online). Thus, our analyses on taxa from extant jawed vertebrate lineages suggest that one of the major MHC paralogs at 2R may have corresponded to a linked huMHC*para-9/12/14* dating back to the jawed vertebrate ancestor but independently broken into multiple translocated fragments throughout gnathostome evolution. A link between the equivalent minor MHC*para-12* and *-14* was also established by Ohta et al. (2019), based on studies of the amphibian *Xenopus* genome.

### A Proto-MHC/AgR Emerged at 1R

Comparative genomics of the MHC region across elasmobranchs and basal bony vertebrates point to a revised scenario of MHC evolution associated with the 2 rounds of WGD. Based on our results, no MHC-I/II genes were detected outside the equivalent huMHC*-6* in elasmobranchs suggesting that MHC genes plus *β2m*, *tap1/2*, and *psmb8,9,10* were present in a single chromosome at 2R in the jawed vertebrate ancestor. A second ancestral chromosome at 2R would include the Ig/TCR genes plus NKC genes, possibly in addition to the three constitutive proteasome genes (*psmb5,6,7*), corresponding to joined huMHC*para-9/12/14* (Fig. 5). These 2 ancestral chromosomes include sets of genes directly associated with adaptive and innate immune responses and share evolutionarily similar genes: C1-type IgSF genes (i.e. found in MHC and Ig/TCR genes), proteasome (constitutive vs. immune), transporter (*tap1/2* vs. *ATP-binding cassette subfamily A member 2* [*abca2*]), and complement genes (c*4* vs. *c5*). Thus, we propose that MHC and Ig/TCR genes present in the equivalent huMHC*para-6* and *para-9* descend from a common ancestral chromosome at 1R, the proto-MHC/AgR (Fig. 5). A close relationship between major *MHCpara-6* and *-9* was previously proposed by Kasahara et al. (1996) based on the presence of duplicated genomic segments in human and mouse and by Katsanis et al. (1996) based on paralogous relationships between human MHC*-6* and MHC*para-9* regions and between MHC*para-19* and MHC*para-1* regions.

**Fig. 6.**
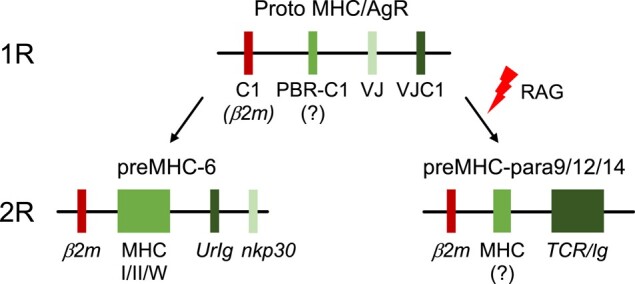
Origin of rearranging and nonrearranging antigen receptors (AgRs) from a proto-MHC/AgR region at 1R. Precursor genes of AgRs of the type VJC1-IgSF were present at 1R after the split between agnathans and proto-gnathostomes. Only one of the descendant chromosomes (MHC-para9/12/14) was invaded by the RAG transposon leading to rearranging AgRs such as Ig and TCR genes. Its sister chromosome, i.e. the precursor of MHC-6, bears nonrearranging AgRs like *UrIg* and *NKp30*. Precursors of MHC genes (PBR-C1) and *β2m* may have already been present at 1R.

A proto-MHC/AgR would have included the major building blocks of adaptive immunity in the same ancestral region, notably *psmb5,6,7*-like genes, a *c3*-like gene, genes encoding presplit variable and joining elements (V and VJ) as well as precursors of the MHC PBR, plus genes comprising single C1-type IgSF domains (C1) (Fig. 5). The latter comprise the minimum essential genetic precursors of AgR (Ig/TCR) and MHC genes. Precursor genes for TAP, i.e. transporters of the ABC superfamily, are likely to have integrated this genomic block (Kasahara et al. 1996). Such genomic architecture at 1R could have allowed early coevolution and/or coregulation among genes and possibly trigger their concerted function (Kaufman 2013). It may have also allowed the assemblage of these building blocks into ancestral MHC and AgR genes, eventually leading to antigen processing, presentation, and receptor genes in the descendant chromosomes (Rogers et al. 2005; Flajnik and Kasahara 2010; Kaufman 2010; Walker et al. 2011). We further speculate that only one of the descendant chromosomes from the proto-MHC/AgR, i.e. equivalent to a linked MHC*para-9/12/14*, would have been subjected to *recombination-activating gene* (*rag*) transposon activity post-2R, leading to split VJ exon and rearranging AgR genes from the common precursor genes in the proto-MHC/AgR (Fig. 5; Chen et al. 2018; Fu et al. 2019). The assemblage of Ig/TCR genes in the proto-MHC/AgR was previously suggested by the presence of VJ genes in the MHC (e.g. *NKp30* and *PRARP*), but this hypothesis has recently been further supported by the discovery of a novel nonrearranging (VJ-C1) Ig-like gene, *UrIg*, in the class III region of the elasmobranch MHC (Flajnik et al. 2023). Thus, the presence of genes with nonsplit V exon in both descendant chromosomes of the proto-MHC/AgR places inception of AgR genes at 1R but postdating the split between jawless and jawed vertebrates (Fig. 6), as no MHC or Ig/TCR genes have been found in agnathans.

The origin and location of *β2m* is not entirely clear from our data but may have already been present at 1R and in both descendant chromosomes (Fig. 5). Indeed, closer examination of *β2m* location and its neighboring genes in basal bony fish suggests such a hypothesis. For instance, *β2m* is found between genes associated with the equivalent huMHC*para-14* (i.e. *IgH* and *TCRαδ*) in bowfin and opossum (Belov et al. 2007; Thompson et al. 2021). In turn, we found *β2m* of reedfish mapping next to genes in MHC*para-12*, namely *tapbpl* and *c*-type lectins (supplementary file S6, Supplementary Material online). Mouse *β2m* is encoded on chromosome 2, the equivalent huMHC*para-9*, but far from the canonical MHC paralogous syntenic group (Michaelson 1981, 1983). This suggests that *β2m* was present in the proto-MHC/AgR at 1R and was differentially silenced on the descendent chromosomic regions depending on the jawed vertebrate lineage and then translocated out during bony fish evolution. In summary, our genomic analyses on elasmobranchs definitively demonstrate ancestral linkage of *β2m* to the MHC, but further study is required to determine whether it arose at 1R or 2R; i.e. it may be the ancestral MHC gene.

### The “0 Hour”: Evolution of the MHC from a PIC

A PIC at 0R has been outlined previously as including framework genes of *rxr*, *neurogenic locus notch homolog protein* (*notch*), *pre-B-cell leukemia homeobox* (*pbx*), and *brd*, in addition to *psmb5,6,7*-like genes and complement *c3-*like as found in cephalochordates (i.e. amphioxus) (Rached et al. 1999; Abi-Rached et al. 2002; Castro et al. 2004; Danchin and Pontarotti 2004). Additionally, genes encoding the VJ and C1-type domains may have also been present at 0R in the vertebrate ancestor. Indeed, genes with VJ and C1-like IgSF domains have been identified in protochordates, and it has been hypothesized that simple exon shuffling would provide the means to create the typical VJ-C1-IgSF structure of AgR genes (Kasahara et al. 2004). Several such VJ-C1-type genes have been identified in tetrapods, some of which are linked to MHC paralogous regions (Kasahara et al. 1996; Ohta et al. 2019; Flajnik et al. 2023) and are perhaps remnants of the original VJ and C1-type precursors in the PIC (Fig. 5).

### Concluding Remarks

The availability of WGAs for understudied basal jawed vertebrates has opened the way to comparative genomic approaches aiming at reconstructing the genomic architecture associated with key events in vertebrate evolution, such as the origin of the AIS. Cartilaginous fish are the oldest extant vertebrate lineage with adaptive immunity and, thus, a key taxon to decipher the original immune gene composition in the gnathostome ancestor and to reconstruct the PIC including the major components of vertebrate adaptive immunity. Using high-quality elasmobranch genomes, we have reconstructed a stable and highly conserved core adaptive MHC region in addition to its sister relationship to the Ig/TCR region in the gnathostome ancestor at 2R. These 2 ancestral chromosomes may have descended from a common proto-MHC/AgR region at 1R bearing the major building blocks of antigen processing, presentation, and receptor genes. While high-quality WGAs are accumulating for elasmobranchs, their more ancient relatives—the Holocephalans—are still a data-deficient taxon. Thus, it remains to be seen if future chromosome-level assemblies on holocephalans will corroborate our current results and hypotheses shown here or whether they will provide additional missing links in the evolutionary history of vertebrate adaptive immunity.

## Materials and Methods

### Genome Mining

WGAs of elasmobranch taxa were surveyed for genes present in the human MHC (chromosome 6) plus hallmark genes of the remaining MHC paralogous regions sensu Ohta et al. (2019), aiming at reconstructing the major and minor MHC paralogous regions in cartilaginous fish. For this purpose, only fully annotated, high-contiguity, chromosome-level WGA of representatives from the 2 sister lineages of elasmobranchs available on NCBI in December 2021 were included, namely for sharks: white shark *C. carcharias* sCarCar2 (GCA_017639515.1, released in May 2021), and catshark *Scyliorhinus canicula* sScyCan1.1 (GenBank GCA_902713625.1, released in January 2021); and for rays: thorny skate *Amblyraja radiata* (GCA_010909765.2, released in April 2021). Upon preliminary inspection, *A. radiata* was not included in downstream analyses as many key genes in reconstructing the MHC region (e.g. *tap*, *c4*, and MHC) were found in unplaced scaffolds and thus not amenable for its robust reconstruction. While preparing this manuscript, another WGA from a ray, i.e. sawfish *P. pectinata* sPriPec2.1 (GCA_009764475.2, released in February 2023), became available and was thus included in the data set. Furthermore, the WGA of the holocephalan *C. milii* IMBC_Cmil_1.0 (GCA_018977255.1, released in July 2021) was included in the analysis, whenever possible given its highly fragmented condition.

Additional WGA of bony fish representatives from basal ray-finned (actinopterygians) and lobe-finned (sarcopterygians) fish lineages were included in the analysis and compared for gene composition and genomic architecture of MHC paralogs across jawed vertebrates. Specifically, ray-finned fish taxa are represented by reedfish *E. calabaricus* fErpCal1.3 (GCA_900747795.4, released in February 2023) while lobe-finned fish taxa included lungfish *P. annectens* PAN1.0 (GCA_019279795.1, released in October 2021) and human *Homo sapiens* GRCh37.p13 (GCA_000001405.14, released March 2022). Gene annotations were checked against the NCBI protein database using *blastp* searches and manually corrected, where applicable, except for *C4* genes in elasmobranchs that were annotated sensu Nonaka et al. (2017). Dedicated synteny analyses were performed for *W*-type MHC genes, *psmb6*, *psmb10*, and *β2m* on the selected elasmobranchs and bony fish genomes (where applicable).

### Bioinformatic Searches

Dedicated bioinformatic searches were conducted to detect MHC-I and MHC-II genes across the surveyed elasmobranch genomes. Query amino acid sequences for all class I (alpha 1 to 3 domains) and class II (alpha 1 to 2 and beta 1 to 2 domains) genes previously reported for cartilaginous fish (i.e. classical and nonclassical; supplementary file S10, Supplementary Material online) were used in *blastx* searches using an *e*-value of <0.001 and query coverage of >50% as cutoffs and identifying the chromosome location of each hit. All hits were checked manually and retrieved both class I and II genes and W-type MHC genes for *e*-values of <10^−08^.

### Phylogenetic Analysis

Protein sequences of putative *W*-type MHC genes retrieved from the surveyed elasmobranch genomes (as described above) were downloaded from NCBI. Reference sequences for *W*-type genes obtained for elasmobranchs (*Triakis scyllium*), coelacanth, and zebrafish from Okamura et al. (2021) (in their supplementary table S3, Supplementary Material online), as well as reference sequences for MHC-Ia and MHC-II alpha and beta genes from elasmobranchs and humans (GenBank accession nos. in supplementary fig. S5, Supplementary Material online) were used in protein alignments in Geneious Prime 2023.0.1 (2005 to 2022 Biomatters Ltd.) using the Clustal Omega algorithm. Diagnostic residues for either MHC-Ia, MHC-II, and *W*-type followed those highlighted in Okamura et al. (2021). Gene identification was further assessed by phylogenetic analysis of the above MHC genes using the neighbor-joining algorithm (Saitou and Nei 1987) and *p*-distances as implemented in MEGAX (Kumar et al. 2018), using 1,000 bootstrap replicates with partial deletion.

## Supplementary Material

msad262_Supplementary_Data

## Data Availability

All data used here were taken from the National Center for Biotechnology Information (NCBI) public database. Gene annotations in chromosomes from the different target taxa are shown in supplementary files S1 to S9, Supplementary Material online including accession numbers, genomic coordinates, and locus number. Accession numbers for genes, sequences, and scaffolds/chromosomes in supplementary figures, Supplementary Material online are shown embedded in the figures.
