# Supplementary material for: An Ancestral Major Histocompatibility Complex Organization in Cartilaginous Fish: Reconstructing MHC Origin and Evolution
Source: Mol Biol Evol. 2023 Dec 6;40(12):msad262. doi: 10.1093/molbev/msad262 (PMC10751288; doi:10.1093/molbev/msad262)
Supplement: msad262_Supplementary_Data [file msad262_supplementary_data.zip › Supplemental Tables and Figures.pdf]

Supplemental Table 1 – Chromosomal locations of genes in the MHC paralogous regions of sharks (white shark *Carcharodon carcharias*, catshark *Scyliorhinus canicula*) and rays (sawfish *Pristis pectinata*).

MHCpara	Hallmark genes	Great White shark *C. carcharias*	Catshark *S. canicula*	Sawfish *P. pectinata*
MHC-6	*brd2*	Chr19: 78,222,535 - 78,242,790	Chr13: 41,455,814 - 41,510,735	Chr34: 13,009,730 - 13,049,337
	notch4	Chr19: 96,549,067 - 96,687,814	Chr13: 17,553,557 - 17,668,737	Chr34: 8,937,158 - 8,951,033
	pbx2	Chr19: 96,831,458 - 96,918,287	*not found*	Chr34: 8,761,764..8,810,758
	rxrb	Chr19: 84,190,244 - 84,284,217	Chr13: 34,776,048 - 34,873,742	Chr34: 15,512,428 - 15,590,812
	psmb8	Chr19: 65,333,720 - 65,350,397	Chr13: 51,796,641 - 51,914,630	Chr34: 17,285,136 - 17,291,615
	psmb9	Chr19: 66,100,434 - 66,217,821	Chr13: 51,742,802 - 51,764,634	Chr34: 17,326,024..17,328,563
	psmb10	Chr15: 112,970,334 - 112,983,876	Chr16: 124,080,744 - 124,098,218	Chr26: 18,672,336 - 18,681,379
	tapbp	Chr19: 112,462,536 - 112,539,191	*not found*	Chr34: 926,051 - 943,038
	c4a	Chr19: 66,732,415 - 66,942,000	*not found*	*not found*
	c4b	Chr19: 88,795,568 - 88,899,866	Chr13: 30,182,677 - 30,288,275	Chr34: 12,377,412 - 12,483,058
MHCpara-1	*brdt*	Chr16: 102,129,958 - 102,207,169	Chr4: 21,038,132 - 21,183,740	Chr3: 14,136,605 - 14,196,011
	nothc2	Chr16: 25,828,373 - 25,991,671	Chr4: 120,335,758 - 120,519,068	Chr3: 63,851,159 - 63,890,712
	pbx1	Chr16: 28,517,654 - 29,066,878	Chr4: 122,003,928 - 122,596,234	Chr3: 64,777,488 - 64,808,497
	rxrg	Chr16: 30,238,807 - 30,594,968	Chr4: 123,298,949 - 123,551,165 (2x)	Chr3: 65,441,830 - 65,465,826
MHCpara-9	*brd3*	Chr8: 143,921,670 - 143,974,262	Chr21: 69,815,527 - 69,849,661	Chr23: 19,777,893 - 19,799,485
	notch1	Chr8: 148,185,406 - 148,285,841	Chr21: 68,134,650 - 68,207,630	Chr23: 18,682,943 - 18,724,966
	pbx3	Chr8: 168,183,620 - 168,402,913	Chr21: 48,796,180 - 49,042,531	Chr23: 6,349,072 - 6,414,574
	rxra	Chr8: 144,117,975 - 144,227,821	Chr21: 69,450,739 - 69,536,856	Chr23: 19,564,378 - 19,584,653
	psmb7	Chr8: 170,893,661 - 170,964,760	Chr21: 45,507,578 - 45,577,624	Chr23: 4,677,473 - 4,740,513
	c5	Chr8: 161,897,282 - 162,052,659	Chr21: 54,329,641 - 54,523,074	Chr23: 9,892,451 - 10,008,186
MHCpara-19	*brd4*	Chr30: 19,132,043 - 19,303,907	Chr25: 15,234,830 - 15,451,189	Chr31: 6,343,244 - 6,476,154
	notch3	Chr30: 11,694,010 - 11,852,261	Chr25: 7,895,147 - 8,083,408	Chr31: 7,214,861 - 7,260,228
	pbx4	Chr30: 26,905,707 - 27,279,572	Chr25: 21,044,245 - 21,437,083	Chr31: 19,232,674 - 19,234,056
	psmb6	Chr33: 995,472 - 1,005,943	*not found*	Chr37: 14,443,637 - 14,478,396
	c3	Chr30: 11,038,045 - 11,205,562	Chr25: 6,701,833- 7,324,024 (3x)	Chr31: 8,410,746 - 8,506,861 (3x)
MHCpara-14	*psmb5*	Chr27: 6,298,442 - 6,302,052	Chr17: 132,000,406 - 132,004,599	*not found*
	*TCRalpha**	Chr27:8197446-8349539	Un|NW_024055500.1:201083-266741	*not found*
	*TCRdelta**	Un|QUOW01000192.1: 199463-199774	Un|NW_024055500.1: 128421-185450	Chr 12: 55595207-55628372
	*IgHM**	Chr27: 8945355-16188405	CHr 17: 110402599-133540320	Chr 12: 54044447-55453464
MHCpara-12	*tapbpl*	Chr40: 304,735 - 315,598	Chr16: 87,398,281 - 87,416,760	Chr21: 11,165,020 - 11,189,259
	a2m	40|NW_024470857.1: 30,855 - 235,238 (x2)	Chr16: 91,303,202 - 92,194,403 (x3)	Chr46: 131,368 - 200,833
	lag3	Un|NW_024470918.1: 1,879 - 22,262	Chr16: 86,991,184 - 87,061,674	*not found*
	cd4	40|NW_024470852.1: 9,206 - 63,764	Chr16: 86,769,825 - 86,959,221	*not found*

Supplemental Table 2 – Chromosomal locations of major and minor MHC paralogous regions in gnathostomes. Cartilaginous fish –white shark *Carcharadon carcharias*, catshark *Scyliorhinus canicula*, sawfish *Pristis pectinata*, ratfish *Callorhincus milii*; ray-finned fish - reedfish *Erpetoichthys calabaricus*; lobe finned-fish - lungfish *Protopterus annectens*.

	**MHCpara-1**	**MHCpara-19**	**MHC-6**	**MHCpara-9**	**MHCpara-14**	**MHCpara-12**
**Cartilaginous fish**						
White shark *C. carcharias*	chr16	chr30	chr19	chr8	chr27	chr40
Catshark *S. canicula*	chr4	chr25	chr13	chr21	chr17	chr16
Sawfish *P. pectinata*	chr3	chr31	chr34	chr23	chr12	*fragmented*
Ratfish *C. milii*	NW_024704746.1	NW_024704770.1		NW_024704749	NW_024704749	NW_024704755
**Ray-finned fish**						
Reedfish *E. calabaricus*	chr10	chr17	chr1	chr9	chr2	chr9
**Lobe-finned fish**						
Lugnfish *P. annectens*	chr10	chr9	chr9	chr16	chr16	chr8

Supplemental Table 3 - Synteny of *psmb6* genes across gnathostomes. Cartilaginous fish – sawfish *Pristis pectinata* and white shark *Carcharadon carcharias*; ray-finned fish - reedfish *Erpetoichthys calabaricus* and spotted gar *Lepisosteus osseus*; lobe finned-fish - lungfish *Protopterus annectens* and human *Homo sapiens*. Genes shared across taxa are highlighted in [illegible]

Cartilaginous fish		Ray-finned fish		Lobe-finned fish	
Sawfish (chr37)	White shark (chr33)	Spotted gar (chr2)	Reedfish (chr3)	Lungfish (chr7)	Human (chr17)
Pristis pectinata	*Carcharodon carcharias*	*Lepisosteus oculatus*	*Erpetoichthys calabaricus*	*Protopterus annectens*	*Homo sapiens*
		ALOX15 B			
	ACADVL	**RNF167**	ERBB3A	BCL6B	MYBBP1A
	DLG4	**ENO3**	PA2G4A	IGL KAPPA	GGT6
	DNAH2	ZNHIT1	RHEBL1	SLC16A13	SMTNL2
	CCT8	ST8SIA1	LAMTOR4	BCL6B	ALOX15
	EFNB1	CYSLTR2	TRAPPC14	LPP	PELP
	CYSLTR3	SRRT	GAL3ST4	SST1A	ARRB2
	SRRT	EPHB4	MINK1	VMO2R	MED11
	SERP2	DLG4	PLD2	UNCHARACT.	CXCL16
	LRCH3	TNF SF13	GHSR	CASR	ZMYND15
	FBOX24	PARP7	UNCHARAC.	VMO2R	TM4 SF5
[illegible]nd of chromosome]	HES4	KCNA1	UNCHARAC.	CASR	VMO1
CPTP	CPTP	TRAPPC1	RNF217	CPTP	GLTP2
**PSMB6**	**PSMB6**	**PSMB6**	**PSMB6**	**PSMB6**	**PSMB6**
HES7	DLX5	GP1BA	SLITRK6	VMO2R (X2)	C17ORF114
CEH31	**CAMTA2**	**KIFC1**	SLC25A11	PLD2	PLD2
HOXA39	**SPAG7**	**CAMTA2**	MSPD1	MINK1	MINK1
**RNF167**	**ENO3**	MOSPD3	**KIF1C**	HSD17B12 (X2)	CHRNE
PRH1	**PFN1**	**SPAG7**	INCA1	EIF4E2	GP1BA
ALOX15 B	**RNF167**	B3GNT7	**CAMTA2**	CHRNG	SLC25A11
**ENO3**	UNCHARAC.	NLGN2	**SPAG7**	SLC25A11	**RNF167**
**SPAG1**	[end of chromosome]	TMEM256	**ENO3**	**RNF167**	**PFN1**
**CAMTA**		TMEM102	**PFN1 (x2)**	**PFN1**	**ENO3**
**KIF1C**		FGF11	**RNF167**	**CAMTA2**	**SPAG7**
PCOLCE		CHRNB1	PCOLCE	**SPAG7**	**CAMTA2**
ZMYND15		CHRNB4	PAGR9	UNCHARACT.	INCA1
				**ENO3**	**KIF1C**
				**PFN1**	SLC52A1
				**KIF1C**	

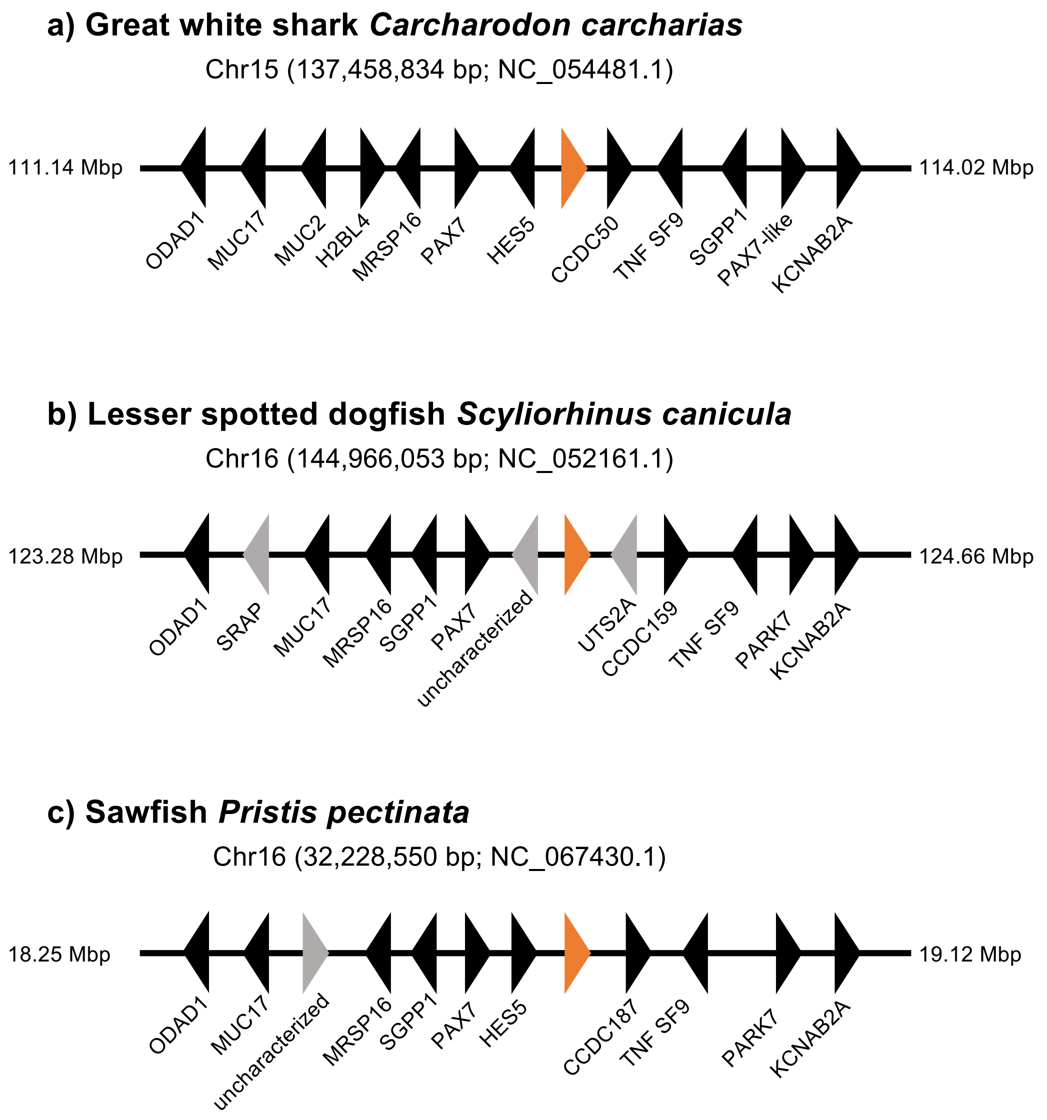

Supplemental Figure 1 – Detailed genomic location of *psmb10* genes (colored orange) in elasmobranchs showing conserved syntenic genes (colored black). Gene size and distances are not to scale. Chromosome length (in base pairs, bp)and GenBank accession no. are shown in parenthesis for each species.

Holocephalan *Callorhincus milii*
(IMBC_Cmil_1.0)

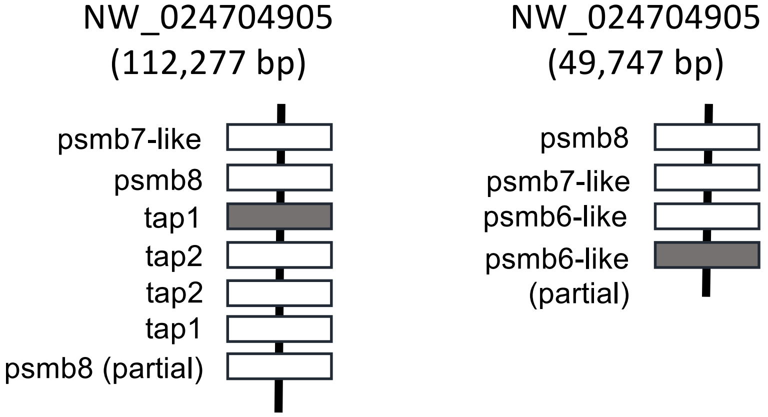

Supplemental Figure 2 – Gene composition of Class I region genes in two scaffolds of the holocephalan *Callorhinchus milii*. GenBank accession no.s are shown on top of each diagram with scaffold length (in base pairs, bp) in parentheses. Genes in grey are pseudogenes. Gene size and distances are not to scale.

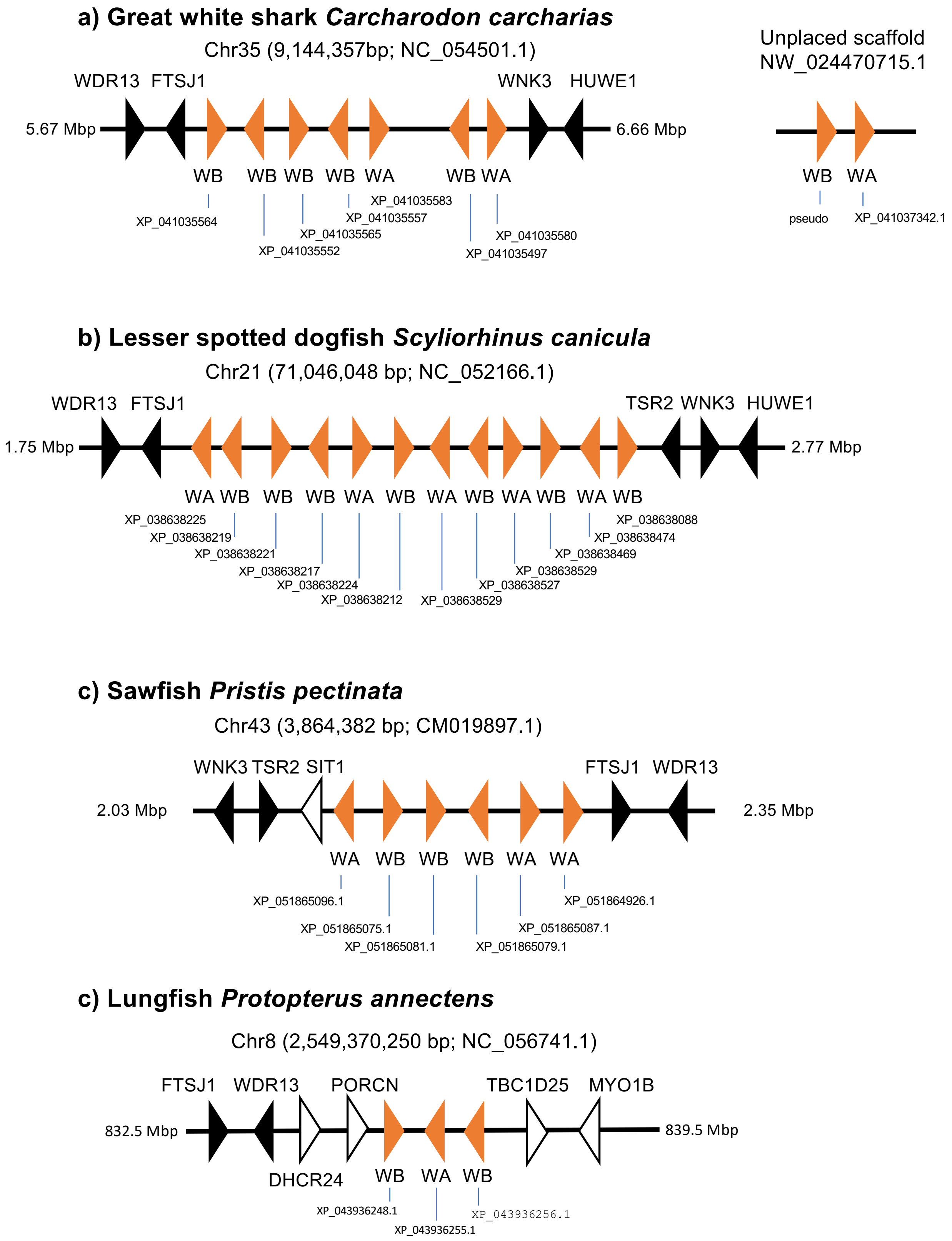

Supplemental Figure 3 – Detailed genomic location of W-type genes (in orange) in non-MHC paralogs of Elasmobranchs (a to c) and the lungfish *P. annectens* (d), showing the shared (in black) and unique (white) syntenic genes among species. GenBank accession numbers for W-type genes are also shown for each species.

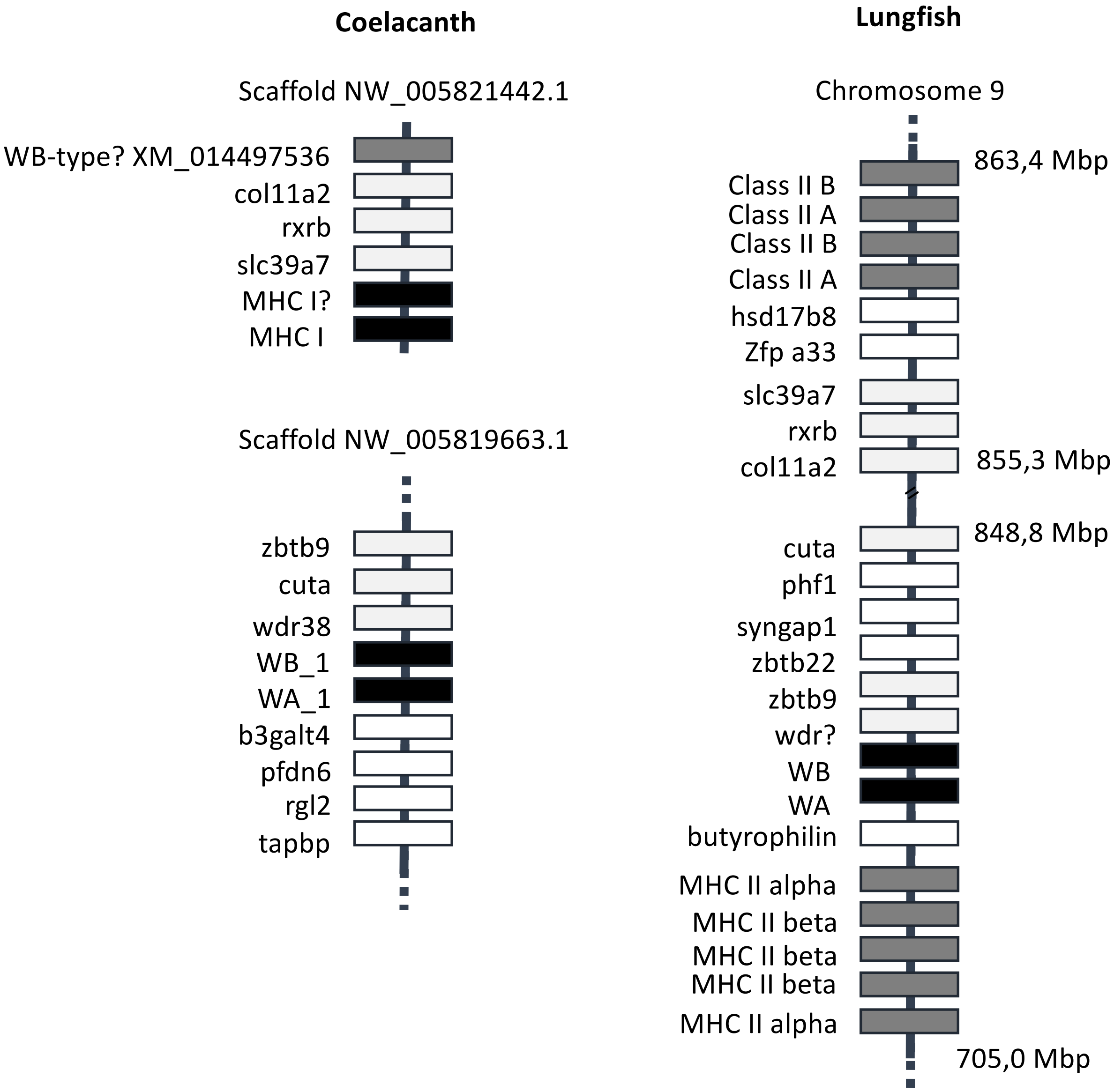

Supplemental Figure 4 - Genomic location of the W-type genes in the MHC of the coelacanth *Latimeria chalumnae* and the lungfish *Protopterus annectens* (colored in black), and their flanking genes (colored white if framework genes, or grey if classical MHC-II). W-type alpha and beta genes of coelacanth follow annotations by Okamura et al. (2021). Gene annotations and genomic coordinates of lungfish are shown in Supplemental File 5.

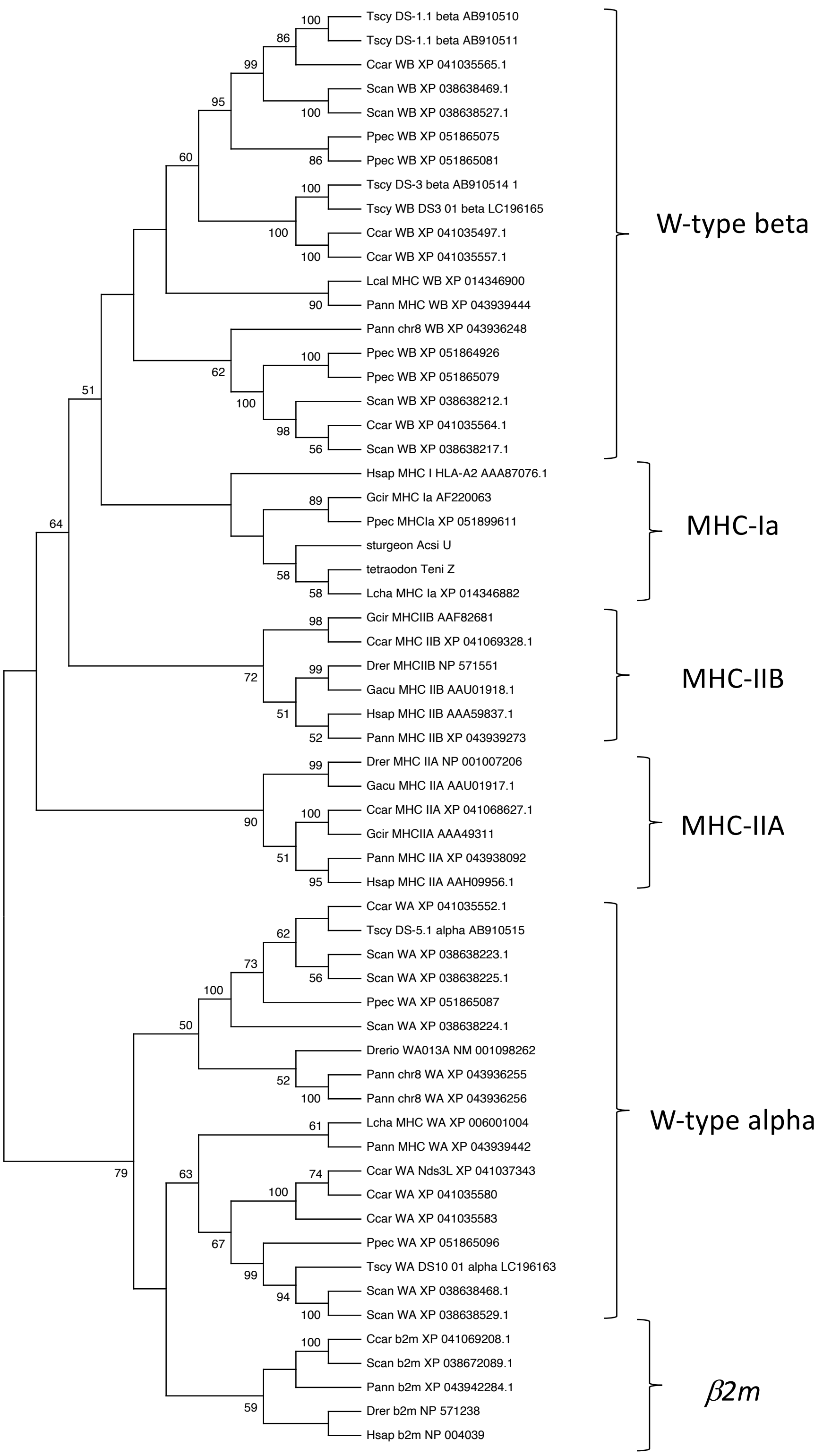

Supplemental Figure 5 - Neighbour-joining phylogenetic tree (midpoint rooting) of W-type genes (WA and WB), MHC-Ia (alpha 3 domain only), MHC-II (IIA, alpha 2; IIB, beta 2 domains only) and *β*2m. Bootstrap support values >50% are shown on top of the respective branch. Tip labels are as follows: Ccar – white shark *Carcharodon carcharias;* Gcir – nurse shark *Ginglymostoma cirratum;* Ppec – sawfish *Pristis pectinata;* Scan – catshark *Scyliorhinus canicula;* Tscy – houndshark *Triakis scyllium;* Lcha – coelacanth *Latimeria chalumnae*; Pann – lungfish *Protopterus annectens*; Drer – zebrafish *Danio rerio*; Gacu – stickleback *Gasteroteus aculeatus*; Hsap – human *Homo sapiens.* GenBank accession numbers are also shown in labels.
